# Task Force Consensus on Nosology and Cut‐Off Values for Axial Postural Abnormalities in Parkinsonism

**DOI:** 10.1002/mdc3.13460

**Published:** 2022-05-09

**Authors:** Michele Tinazzi, Christian Geroin, Roongroj Bhidayasiri, Bastiaan R. Bloem, Tamine Capato, Ruth Djaldetti, Karen Doherty, Alfonso Fasano, Houyam Tibar, Leonardo Lopiano, Nils G. Margraf, Marcelo Merello, Caroline Moreau, Yoshikazu Ugawa, Carlo Alberto Artusi, the International Parkinson and Movement Disorders Society Task Force on Postural Abnormalities

**Affiliations:** ^1^ Neurology Unit, Movement Disorders Division, Department of Neurosciences Biomedicine and Movement Sciences University of Verona Verona Italy; ^2^ Chulalongkorn Centre of Excellence for Parkinson's Disease and Related Disorders, Department of Medicine, Faculty of Medicine Chulalongkorn University and King Chulalongkorn Memorial Hospital, Thai Red Cross Society Bangkok Thailand; ^3^ The Academy of Science The Royal Society of Thailand Bangkok Thailand; ^4^ Department of Neurology Radboud University Medical Centre, Donders Institute for Brain, Cognition, and Behavior Nijmegen The Netherlands; ^5^ Department of Neurology, Movement Disorders Center University of São Paulo São Paulo Brazil; ^6^ Department of Neurology Rabin Medical Center; Sackler Faculty of Medicine, Tel Aviv University Tel Aviv Israel; ^7^ Department of Neurology Royal Victoria Hospital Belfast Ireland; ^8^ Centre for Medical Education Queens University Belfast Ireland; ^9^ Division of Neurology University of Toronto Toronto Ontario Canada; ^10^ Krembil Brain Institute Toronto Ontario Canada; ^11^ Edmond J. Safra Program in Parkinson's Disease and Morton and Gloria Shulman. Movement Disorders Clinic Toronto Western Hospital, UHN Toronto Ontario Canada; ^12^ Service de Neurologie B et de Neurogénétique Hôpital des spécialités OTO‐Neuro‐Ophtalmologique Ibn Sina University hospital, Medical school of Rabat, Mohamed 5 University of Rabat Rabat Morocco; ^13^ Department of Neuroscience “Rita Levi Montalcini” University of Turin Torino Italy; ^14^ Neurology 2 Unit A.O.U. Città della Salute e della Scienza di Torino Torino Italy; ^15^ Department of Neurology UKSH, Christian‐Albrechts‐University Kiel Germany; ^16^ Movement Disorders Service FLENI, CONICET Buenos Aires Argentina; ^17^ Expert center for Parkinson's disease, Neurological department, Inserm UMR 1171 Lille University Hospital Lille France; ^18^ Department of Human Neurophysiology, School of Medicine Fukushima Medical University Fukushima Japan

**Keywords:** postural abnormalities, Parkinson's disease, atypical parkinsonisms, camptocormia, Pisa syndrome, antecollis, diagnostic criteria.

## Abstract

**Background:**

There is no consensus with regard to the nosology and cut‐off values for postural abnormalities in parkinsonism.

**Objective:**

To reach a consensus regarding the nosology and cut‐off values.

**Methods:**

Using a modified Delphi panel method, multiple rounds of questionnaires were conducted by movement disorder experts to define nosology and cut‐offs of postural abnormalities.

**Results:**

After separating axial from appendicular postural deformities, a full agreement was found for the following terms and cut‐offs: camptocormia, with thoracic fulcrum (>45°) or lumbar fulcrum (>30°), Pisa syndrome (>10°), and antecollis (>45°). “Anterior trunk flexion,” with thoracic (≥25° to ≤45°) or lumbar fulcrum (>15° to ≤30°), “lateral trunk flexion” (≥5° to ≤10°), and “anterior neck flexion” (>35° to ≤45°) were chosen for milder postural abnormalities.

**Conclusions:**

For axial postural abnormalities, we recommend the use of proposed cut‐offs and six unique terms, namely camptocormia, Pisa syndrome, antecollis, anterior trunk flexion, lateral trunk flexion, anterior neck flexion, to harmonize clinical practice and future research.

Postural abnormalities are motor symptoms complicating the clinical picture of patients with parkinsonism,^1^, ^2^ typically referred to as abnormal spine flexions appearing while sitting or standing, worsened during walking, and usually resolved in the lying position. This is not the case in longstanding conditions or for antecollis in multiple system atrophy, when postural abnormalities may be irreversible or only partially reversible because of the (co)occurrence of musculoskeletal deformities or dystonia.^2^, ^3^, ^4^

Postural abnormalities are frequent (estimated prevalence in Parkinson's disease [PD] ~20% or even higher when considering mild forms),^1^ can occur either in isolated or combined forms, can be accompanied by other musculoskeletal comorbidities like scoliosis^2^ and are associated with an increased prevalence of back pain and falls, higher levels of disability, and reduced quality of life.^1^, ^2^, ^4^, ^5^, ^6^

Despite such remarkable clinical impact, the pathophysiology of postural abnormalities remains largely unknown^2^, ^7^ and the current pharmacological and non‐pharmacological treatment options may at best offer only short‐term and partial improvements.^8^, ^9^ The progress of research in the diagnosis, management, and prevention of postural abnormalities continues to be hampered by two basic issues related to the lack of consensus on nosology and cut‐off values, leading to an extreme heterogeneity in the literature.^7^, ^10^

According to the mandate of the International Parkinson and Movement Disorders Society (MDS) Task Force on Postural Abnormalities in Parkinsonism, we present a consensus study on the nosology and cut‐off values of postural abnormalities.

## Materials and Methods

### Study Design

**FIG 1:**
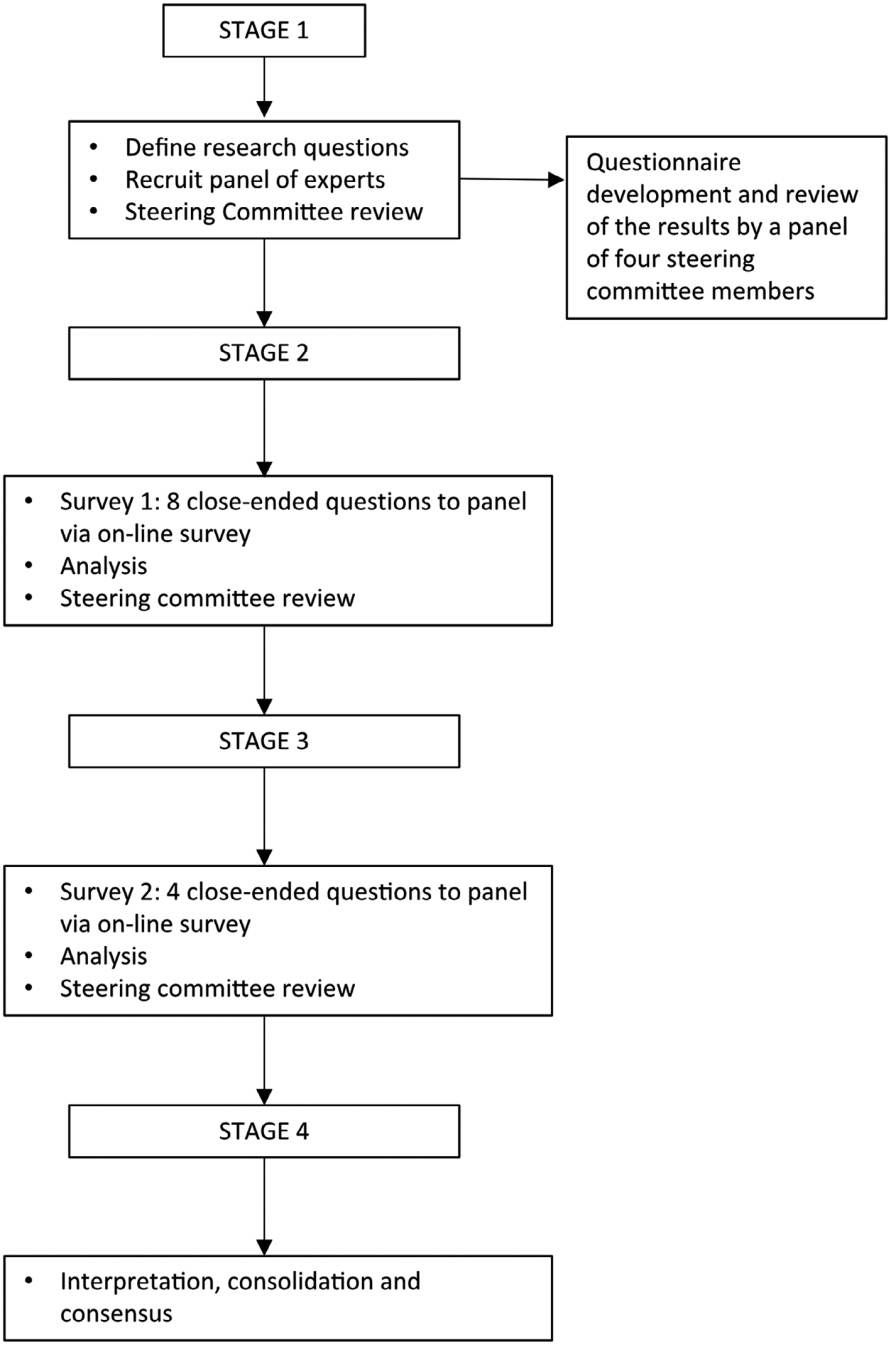
Flow‐chart of the study.

A modified Delphi study was conducted with a panel of international experts belonging to the MDS Task Force on Postural Abnormalities. This method proved to be valid and reliable to achieve convergence of opinion on topics in healthcare that have not been previously examined.^11^, ^12^, ^13^, ^14^ The Delphi methodology is based on multiple rounds of anonymous and structured questionnaires. Eleven movement disorders specialists (neurologists, neurosurgeons, and physiotherapists) were selected to answer questionnaires through several rounds of two different surveys, one related to nosology and the other to cut‐off values of postural abnormalities. Four additional members (M.T., C.G., R.B., and C.A.A.) who belonged to the steering committee organized and distributed the questionnaires and sent to the panelists a personal web survey link created with Google Forms (freeware software) via e‐mail. Disagreements were solved by web‐based meetings to promote an interactive discussion before reaching final consensus. In Figure 1 is the reported flow‐chart of the modified Delphi study.

### Survey 1: Consensus on Nosology

An eight close‐ended question survey was designed to reach consensus on: (1) separating axial from appendicular postural deformities (ie, hand and foot deformities), (2) identifying a common general term for axial non‐physiological postures, (3) confirming the terms antecollis or anterocollis, camptocormia, and Pisa syndrome to define severe axial non‐physiological postures, and (4) identifying the terms describing less severe axial non‐physiological postures (a “grey area” between the classical severe axial posture abnormalities and a normal posture). Additionally, free text entry was allowed to provide terms other than those already enlisted (Table 1).

**TABLE 1:** Questionnaire on Survey 1: nosology

Question	Possible answers
Would you agree to split axial not physiological postures from appendicular ones (ie, hand and foot deformities)?	Yes, no
Which term is the most appropriate to identify axial not physiological postures typical of patients with parkinsonism?	Abnormal postures, postural abnormalities, postural deviations, postural deformities, trunk asymmetry, trunk posture disturbances, trunk deformities, trunk flexion, bent spine, other (free enter)
Would you agree to maintain the term “camptocormia” to indicate a reversible, severe anterior trunk flexion?	Yes, no
Would you agree to maintain the term “Pisa syndrome” to indicate a reversible, severe lateral trunk flexion?	Yes, no
Which one of the two terms you believe is the best to indicate a reversible, severe anterior neck flexion?	Anterocollis, antecollis
Which term is the most appropriate to identify a lateral trunk flexion not severe enough to be called Pisa syndrome?	Side leaning, lateral bending, lateral flexion, lateral trunk deviation, lateral trunk bending, lateral trunk flexion, frontal plane trunk deformity, frontal plane trunk flexion, mild Pisa syndrome, early Pisa syndrome, pre Pisa syndrome, other (free enter)
Which term is the most appropriate to identify an anterior trunk flexion not severe enough to be called camptocormia (independently from the fulcrum of bending)?	Forward bending, anterior trunk deviation, anterior trunk bending, anterior trunk flexion, sagittal plane trunk deformity, sagittal plane trunk flexion, stooped posture, mild camptocormia, early camptocormia, pre camptocormia, other (free enter)
Which term is the most appropriate to identify an anterior neck flexion not severe enough to be called antecollis?	Anterior neck flexion, anterior neck bending, anterior neck deviation, mild antecollis/anterocollis, pre anterocollis/antecollis, other (free enter)

### Survey 2: Consensus on Cut‐Off Values

**FIG 2:**
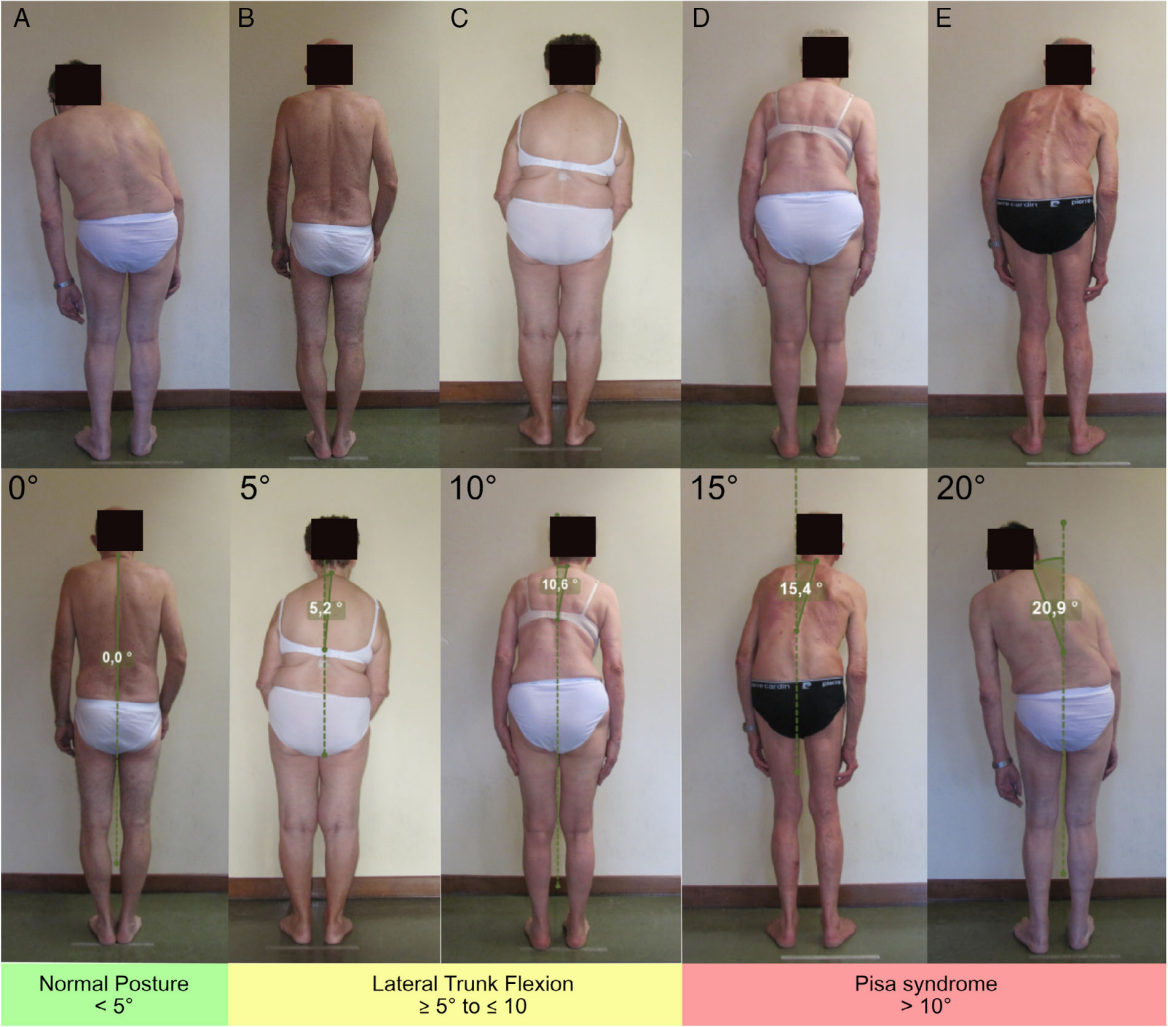
Pictures of patients with PD presenting a variable degree of lateral trunk flexion, measured according to the perpendicular method (bottom line).

**FIG 3:**
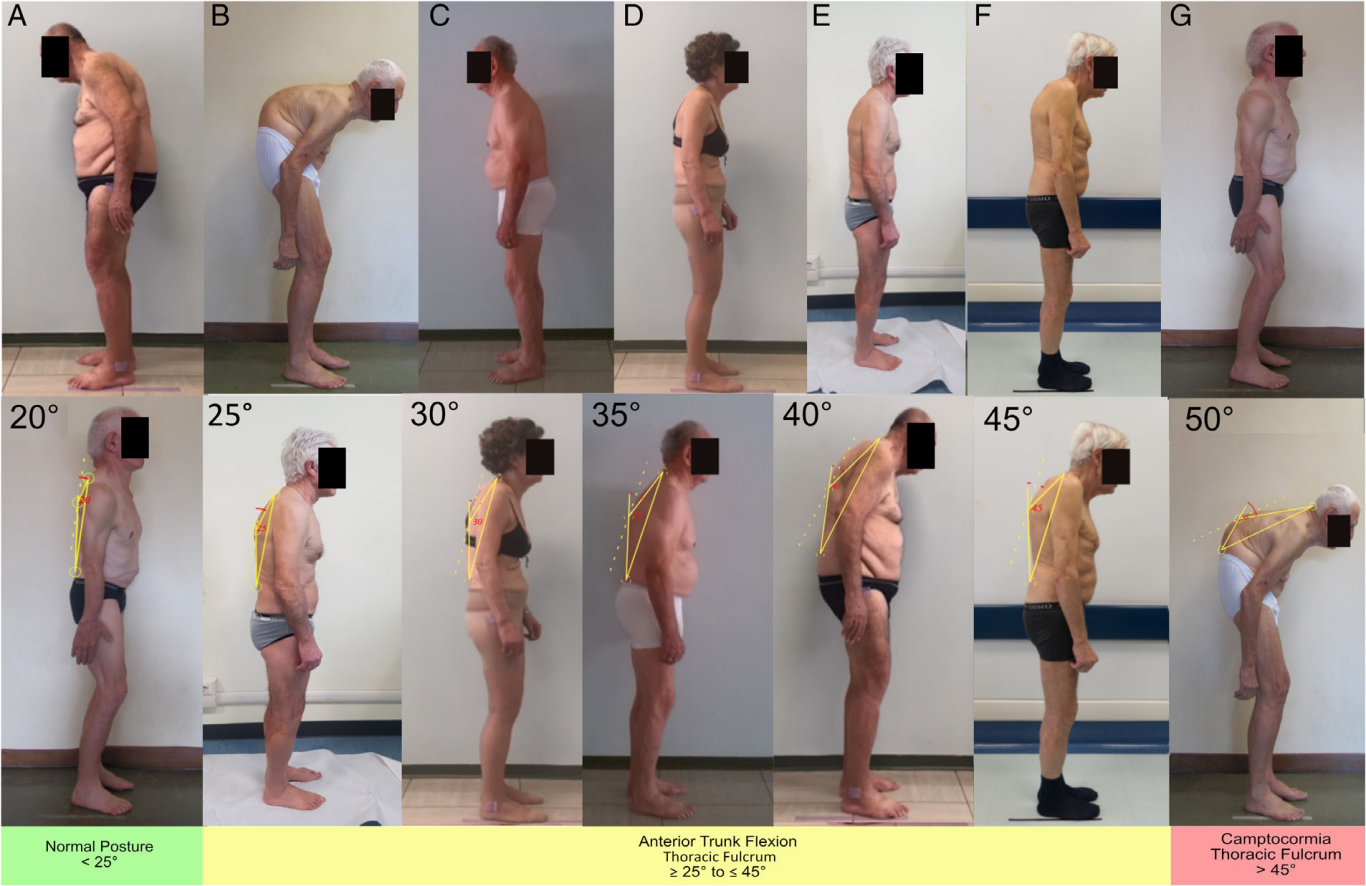
Pictures of patients with PD presenting a variable degree of anterior trunk flexion (thoracic fulcrum), measured according to the upper method (bottom line).

**FIG 4:**
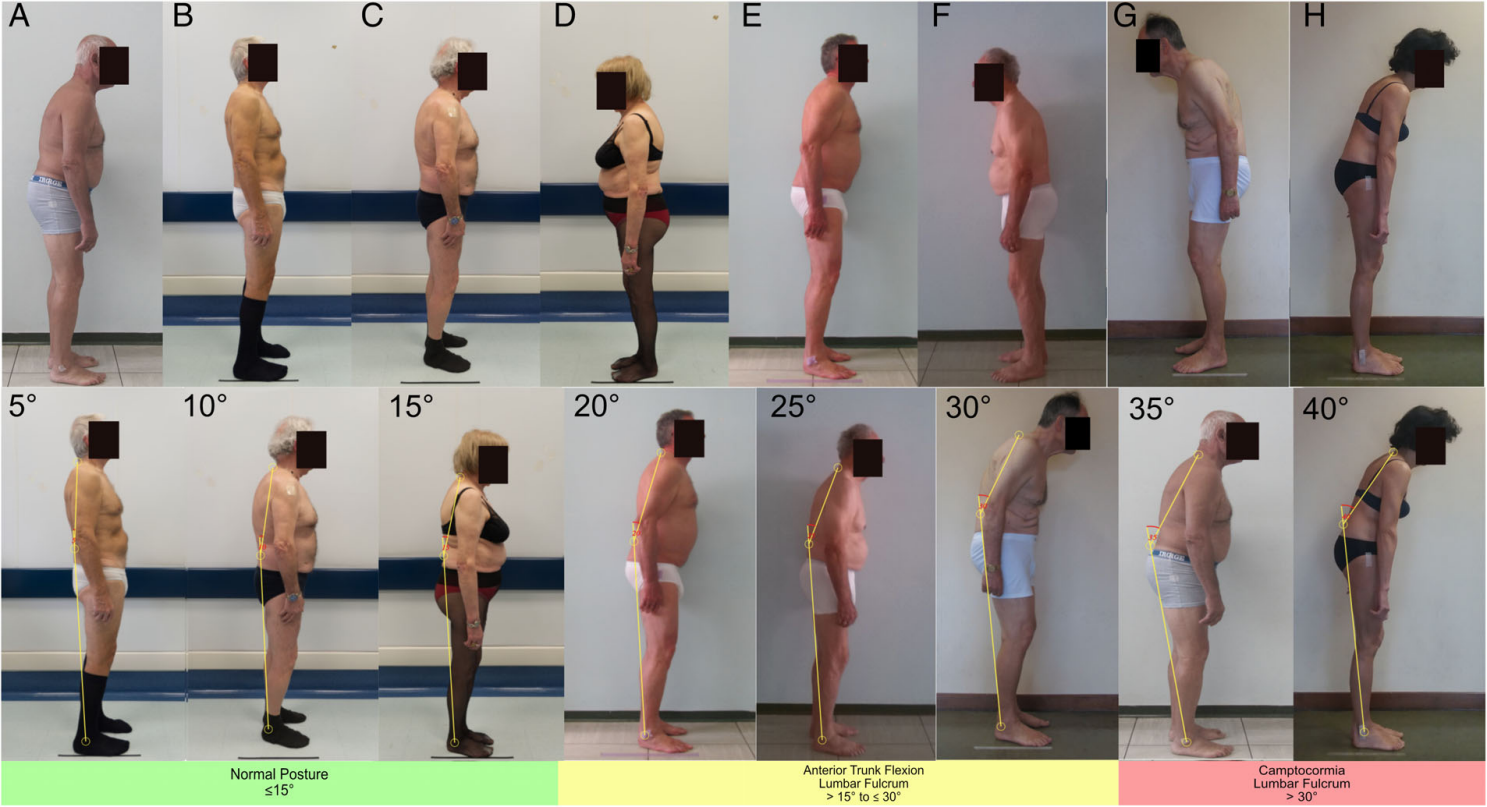
Pictures of patients with PD presenting a variable degree of anterior trunk flexion (lumbar fulcrum), measured according to the malleolus method (bottom line).

**FIG 5:**
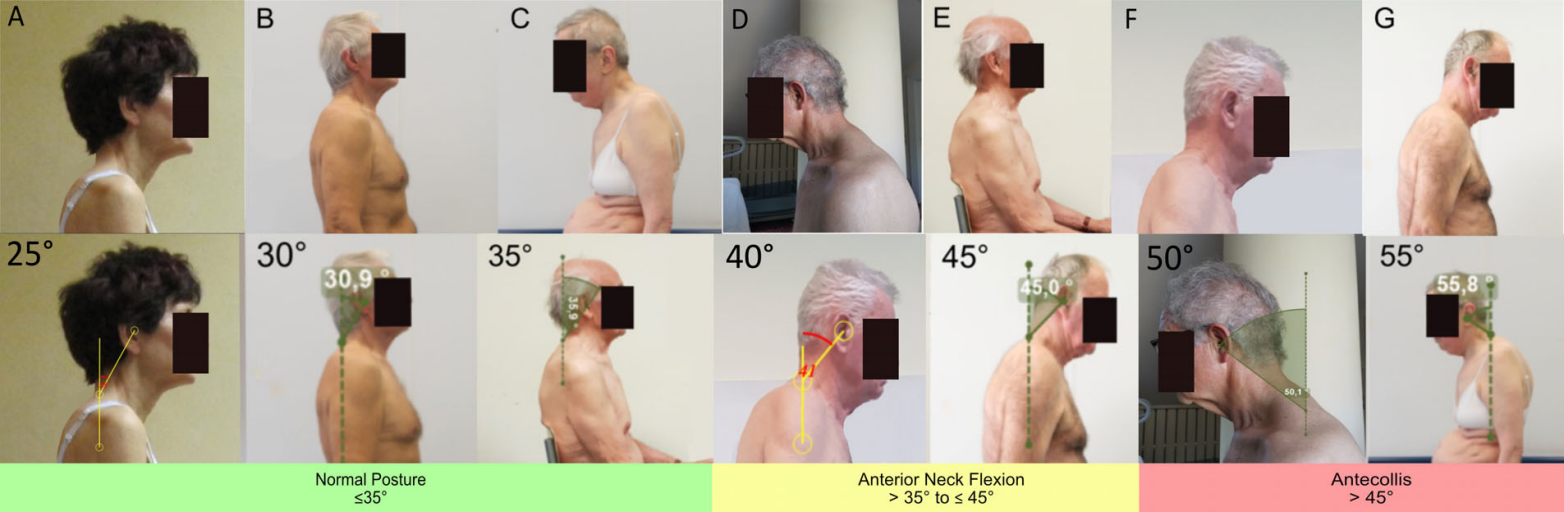
Pictures of patients with PD presenting a variable degree of anterior neck flexion, measured according to the perpendicular method (bottom line).

After reaching a consensus on nosology, we performed a second survey to obtain a consensus on the cut‐off values for the definition of each postural abnormality. We provided pictures showing the lateral or backside view of 26 undressed PD patients with a variable combination of trunk flexion sides and degrees, calculated according to validated software‐based methods (Figs. 2, 3, 4, 5).^15^, ^16^ A detailed description of the patients' pictures is reported in the supplementary material. Diagnoses with complete agreement among experts were taken into account to draft a definition of normal posture, lateral/anterior trunk/anterior neck flexion, Pisa syndrome/camptocormia/antecollis, and later shared and discussed during web‐based meetings by all experts until a definition satisfying all panelists was achieved.

### Data Analysis

Aggregated results obtained from survey 1 and 2 were analyzed, presented, and discussed in a web‐meeting among panelists. For both surveys, a consensus for each question was pre‐defined as ≥70% agreement among the panelists.^14^ In case of disagreements after two rounds, a real‐time and web‐based anonymous polling was performed at the end of the discussion and an agreement consensus was obtained for all remaining questions.

Relevant aspects and issues that emerged during the meeting were recorded and used for a more comprehensive evaluation of the consensus criteria.

## Results

In the timeframe between January and September 2021, all panelists participated in the two surveys: (1) nosology and (2) cut‐off values. The survey on nosology required a total of three rounds before reaching a final consensus on all terms (Table 2). The survey on cut‐off values required a total of three rounds before reaching a final consensus on different cut‐offs (Table 3, Figs. 2, 3, 4, 5). At the end of round 3, the summary of results achieved form the entire process was approved by 100% of panelists and the final consensus for nosology and cut‐offs was reported in Table 2.

**TABLE 2:** Proposed nosology and cut‐off values of postural abnormalities in parkinsonism

Nosology	Agreement
When you evaluate patients with parkinsonism, consider to:	
1) Split axial not physiological postures from appendicular ones (ie, hand and foot deformities)	100%, round 1
2) Use the term “postural abnormalities” to indicate Pisa syndrome, camptocormia, antecollis, and milder forms of axial postural abnormalities	72%, round 1
3) Use the term “camptocormia” to indicate a reversible and severe anterior (sagittal plane) flexion of the trunk, according with its diagnostic criteria	90.9%, round 1
4) Use the term “Pisa syndrome” to indicate a reversible and severe lateral (coronal plane) flexion of the trunk, according with its diagnostic criteria	90.9%, round 1
5) Use the term “antecollis” to indicate a reversible and severe anterior (sagittal plane) flexion of the neck, according with its diagnostic criteria	72.7%, round 2
6) For postural abnormalities not severe enough to be called camptocormia, Pisa syndrome or antecollis, use the following terms: “anterior trunk flexion,” “lateral trunk flexion,” and “anterior neck flexion”	90.9%, round 3 (upper and lower anterior trunk flexion and lateral trunk flexion); 81.8%, round 3 anterior neck flexion)
Cut‐off values	
Coronal plane postural abnormalities	
<5°	Normal posture
≥5° to ≤10°	Lateral trunk flexion
>10°	Pisa syndrome
Sagittal plane postural abnormalities	
Thoracic	
<25°	Normal posture
≥25° to ≤45°	Anterior trunk flexion thoracic fulcrum (C7‐T12 vertebrae)
>45°	Camptocormia thoracic fulcrum (C7‐T12 vertebrae)
Lumbar	
≤15°	Normal posture
>15° to ≤30°	Anterior trunk flexion lumbar fulcrum (L1–L5 vertebrae, hip flexion)
>30°	Camptocormia lumbar fulcrum (L1–L5 vertebrae, hip flexion)
Cervical	
≤35°	Normal neck posture
>35° to ≤45°	Anterior neck flexion
>45°	Antecollis

**TABLE 3:** Agreement on cut‐off values in the different types and degrees of postures

Type of postural abnormality	Degree (°)	Type of posture	Agreement (%)	Round
Lateral trunk flexion (Fig. 2)	0	Normal posture	90.9	1
	5	Lateral trunk flexion	100	1
	10	Lateral trunk flexion	81.8	1
	15	Pisa syndrome	81.8	2
	20	Pisa syndrome	100	1
Anterior trunk flexion (thoracic level) (Fig. 3)	20	Normal posture	100	3
	25	Anterior trunk flexion	72.7	3
	30	Anterior trunk flexion	81.8	1
	35	Anterior trunk flexion	72.7	1
	40	Anterior trunk flexion	100	3
	45	Anterior trunk flexion	81.8	2
	50	Camptocormia	100	1
Anterior trunk flexion (lumbar level) (Fig. 4)	5	Normal posture	100	1
	10	Normal posture	100	1
	15	Normal posture	72.7	1
	20	Anterior trunk flexion	90.9%	1
	25	Anterior trunk flexion	72.7	1
	30	Anterior trunk flexion	100	3
	35	Camptocormia	100	3
	40	Camptocormia	90.9	1
Anterior neck flexion (Fig. 5)	25	Normal posture	81.8	3
	30	Normal posture	81.8	1
	35	Normal posture	90.9	2
	40	Anterior neck flexion	100	3
	45	Anterior neck flexion	81.8	1
	50	Antecollis	100	3
	55	Antecollis	81.8	1

## Discussion

The results of this study provide consensus on the nosology and cut‐off values for postural abnormalities in parkinsonism. We found a full consensus using the terms “anterior trunk flexion,” “lateral trunk flexion,” and “anterior neck flexion” to identify those forms of postural abnormalities not severe enough to be called “camptocormia,” “Pisa syndrome,” and “antecollis,” respectively. We also agreed on the cut‐offs to distinguish between the different forms of postural abnormalities.

Postural abnormalities represent hallmark motor symptoms of PD and atypical parkinsonism^2^; however, evidence‐based guidelines for their management are missing. Advances on diagnosis, management, and prevention of postural abnormalities should be a research priority, but the lack of consensus on nosology and diagnostic cut‐offs have hampered the possibility of obtaining solid evidence in this field, because of an extreme variability in the results of epidemiological, pathophysiology, and interventional studies.^7^, ^10^ The proposed criteria can help to overcome the impressive heterogeneity on postural abnormalities prevalence data with percentages in PD ranging from 3% to 17% for camptocormia,^10^, ^17^ from 2% to 9% for Pisa syndrome,^18^, ^19^ and from 5% to 9% for antecollis.^1^, ^20^ Moreover, a uniformity in nosology and cut‐off values, with the novelty of considering mild forms of postural abnormalities, may facilitate research on treatment and prevention programs. The early recognition of postural abnormalities should foster the prompt establishment of treatment strategies, encompassing medical therapy (eg, dopaminergic medications, deep brain stimulation, and botulinum toxin),^8^ physiotherapy interventions,^9^ and lifestyle changes. All these actions are used to avoid the evolution toward structured, fixed postural abnormalities, leading to severe mechanical constraints affecting respiration, mobility, and postural stability.

To obtain a reliable evaluation of postural abnormalities, patients should be evaluated with adequate exposure of affected body parts and degrees of spine flexion calculated on both coronal and sagittal plane. Degrees of spine flexion and fulcra should be separately assessed, according to the currently accepted evaluation methods.^15^ In particular, we recommend to calculate posture degrees by analyzing photographs using the NeuroPostureApp, a freeware software‐based measurements (https://www.neuroimaging.uni-kiel.de/NeuroPostureApp/).^16^ Wall goniometer or smartphone can be used instead, but possible underestimation of the degrees with these methods should be considered.^15^

Comorbidities should always be carefully assessed when approaching patients with postural abnormalities to exclude the presence of other diseases potentially causing the abnormal posture, with relevant implications in terms of treatment and prognosis.^2^, ^7^ Therefore, an accurate evaluation of orthopedic diseases should be performed, potentially with the help of radiological exams of the spine, and signs or symptoms of primary muscular or neuromuscular junction diseases (eg, myasthenia gravis, muscular dystrophies, structural or inflammatory myopathies), motor neuron diseases, and rheumatic inflammatory diseases (eg, ankylosing spondylitis) should be excluded.

Although the commonly accepted definitions of camptocormia and Pisa syndrome currently encompass the possibility of trunk realignment by passive mobilization or when the patient is lying on the bed, the reversibility should not be a core diagnostic criterion for a diagnosis of postural abnormality, because over time patients with postural abnormalities tend to progress toward a fixed, irreversible spine misalignment (eg, the common appearance of scoliosis with irreversible vertebrae rotation in patients with long‐lasting Pisa syndrome).^21^ However, we acknowledge that, especially when the postural abnormalities onset is recent, the presence of irreversible postural abnormalities should be considered a red flag for an alternative diagnosis. We recommend the presence or absence of postural abnormalities reversibility is always assessed and reported by evaluating the patient in a supine position on a bed because it is relevant in both prognosis and management.

Although patient's awareness of postural abnormalities may be low,^22^ pain, imbalance, and limitations in daily life activities may derive from postural abnormalities, and these aspects should be investigated. To date, a formal, validated scale evaluating the impact of postural abnormalities in parkinsonism is still missing and represents a research gap. Patient‐centered outcome measures are warranted to better analyze the impact of postural abnormalities on subjects and the efficacy of therapeutic approaches.

A limitation of the Delphi approach is that it may represent a compromise position (or a middle‐of‐the‐road consensus) because of a tendency to eliminate extreme positions. The multiple rounds structure aimed to reach a final consensus, inevitably leads experts to reconsider (and possibly revise) their previous position and answers in light of the responses of other panelists. Moreover, pictures evaluation relied on subjective visual estimation of postural abnormalities, which can vary among raters according with their experience. Moreover, the cut‐offs of postural abnormalities generated by this consensus do not necessary reflect a clinical significant difference for patients nor pathophysiological differences and this aspect must be addressed using appropriate scales in future studies.^7^ In the absence of strong evidence on the pathophysiology of postural abnormalities and, specifically of camptocormia, this consensus was in favor of considering camptocormia as a single entity, but differentiating the spine level of the fulcrum in thoracic and lumbar, which can be useful for therapeutic purposes. Indeed, electromyographical studies suggested the presence of hyperactivity in different muscles in the two forms, deserving targeted interventions.^10^

In conclusion, we have provided specific terms and cut‐offs values for the diagnosis of postural abnormalities in patients with PD and other parkinsonism. Taking into account the arbitrary nature of the choices made by the expert consensus, the diffusion of these nosology and cut‐off values should: (1) provide clinicians a shared language and favor an earlier identification of patients with postural abnormalities for referral to specialized centers for the best management, and (2) improve the uniformity of inclusion criteria in research studies.

## Author Roles

(1) Research project: A. Conception, B. Organization, C. Execution; (2) Statistical Analysis: A. Design, B. Execution, C. Review and Critique; (3) Manuscript: A. Writing of the First Draft, B. Review and Critique.

M.T.: 1A, 1B, 1C, 2A, 2B, 2C, 3A, 3B

C.G.: 1A, 2B, 2C, 2A, 2B, 2C, 3A, 3B

R.B.: 1A, 1B, 1C, 2A, 2C, 3B

B.R.B.: 1C, 2C, 3B

T.C.: 1C, 2C, 3B

R.D.: 1C, 2C, 3B

K.D.: 1C, 2C, 3B

A.F.: 1C, 2C, 3B

H.T.: 1C, 2C, 3B

L.L.: 1C, 2C, 3B

N.G.M.: 1C, 2C, 3B

M.M.: 1C, 2C, 3B

C.M.: 1C, 2C, 3B

Y.U.: 1C, 2C, 3B

C.A.A.: 1A, 1B, 1C, 2A, 2B, 2C, 3A, 3B

## Disclosures

**Ethical Compliance Statement:** The institutional review board of the Azienda Ospedaliera Universitaria Integrata Verona reviewed and approved the study (approval number Prog.1655CESC). All patients (or their guardians) were informed about the nature of the study and gave their written consent to participate (consent for research). Authorization has been obtained for disclosure (consent to disclose) of any recognizable persons in photographs. We confirm that we have read the Journal's position on issues involved in ethical publication and affirm that this work is consistent with those guidelines.

**Funding Sources and Conflicts Of Interest:** No specific funding was received for this work. The authors declare that there are no conflicts of interest relevant to this work.

**Financial Disclosures for the Previous 12 Months:** R.B. receives salary from Chulalongkorn University and stipend from the Royal Society of Thailand, has received consultancy and/or honoraria/lecture fees from Abbott, Boehringer‐Ingelheim, Britannia, Ipsen, Novartis, Teva‐Lundbeck, Takeda, and Otsuka pharmaceuticals; he has received research funding from the Newton Fund, the United Kingdom (UK) Government, Thailand Science and Research Innovation Bureau, Thailand Research Fund, Crown Property Bureau, Chulalongkorn University, and the National Science and Technology Development Agency; he holds patents for laser‐guided walking stick, portable tremor device, nocturnal monitoring, and electronic Parkinson's disease symptom diary as well as copyright on Parkinson's mascot, dopamine lyrics, and teaching video clips for common nocturnal and gastrointestinal symptoms for Parkinson's disease. A.F. has received consultancies from AbbVie, Medtronic, Boston Scientific, Sunovion, Ipsen, Merz; Advisory Boards of AbbVie, Boston Scientific, Ceregate, Inbrain, Ipsen, Medtronic, Jazz, and Biogen/Sage; he has received honoraria from AbbVie, Medtronic, Boston Scientific, Sunovion, UCB, and Ipsen; he has received grants from University of Toronto, Weston foundation, AbbVie, Medtronic, Boston Scientific, the Michael J. Fox Foundation, European Union, MSA coalition, and Dystonia Medical Research Foundation. L.L. has received consultancies and honoraria from Bial, Zambon, and AbbVie; and grants from University of Turin, Zambon, and AbbVie. N.M. is on the advisory boards for Angelini, and GW Pharmaceuticals; and has received grants from UCB Pharma, Desitin, Eisai, LivaNova, Angelini, and GW Pharmaceuticals. M.M. is a consultant for St. Jude/Abbott; he has received honoraria from Glaxo; he has research grants from Glaxo, Allergan, and TEVA; he has received royalties from Springer, Random House, Cambridge University press, and Humana Press; and is an Editor honorarium at Wiley and Son, Movement Disorders Society.

## Supporting information


**Supplementary Material**: Detailed methods for the consensus on cut‐off values.
