# Supplementary material for: Task Force Consensus on Nosology and Cut‐Off Values for Axial Postural Abnormalities in Parkinsonism
Source: Mov Disord Clin Pract. 2022 May 9;9(5):594–603. doi: 10.1002/mdc3.13460 (PMC9274349; doi:10.1002/mdc3.13460)
Supplement: Supplementary file 1 — Supplementary Material: Detailed methods for the consensus on cut‐off values. [file MDC3-9-594-s001.docx]

**Supplementary material**

**Detailed methods for the consensus on cut-off values**

Five pictures with 0° to 20° of flexion were provided to panelists for the lateral trunk flexion, 7 pictures with 20° to 50° for the anterior trunk flexion at the thoracic level, 8 pictures with 5° to 40° for the anterior trunk flexion at the lumbar level, and 7 pictures with 25° to 55° for anterior neck flexion. The choice of starting from 20° of thoracic trunk flexion and 25° of neck flexion was due to the fact that these angles are physiological and represent normal spine curvatures in healthy adult people (Schlenstedt et al., 2020). All patients presented a flexion of the cervical, thoracic or lumbar spine, which appeared during standing or walking. No patient had coexistent neurological diseases known to negatively affect posture, previous major spinal surgery, skeletal and/or muscle disease (e.g., vertebral fractures, spondylodiscitis, inflammatory myopathy) or treatment with medications possibly leading to PA (neuroleptics other than clozapine or quetiapine and antiemetics, except for domperidone) in the 6 months prior to enrolment.

Pictures of patients were displayed randomly and panelists were asked to judge each photograph (blinded for the calculated degrees of flexion) and decide for each photo the presence and type of a specific postural abnormality, choosing between: no postural abnormalities, Pisa syndrome/camptocormia with thoracic fulcrum/camptocormia with lumbar fulcrum/antecollis or less severe axial not physiological postures with misalignment of the neck and of the trunk in the sagittal and coronal plane, according to the definitions achieved by the survey on terminology.

**Reference**

Schlenstedt, C., Bosse, K., Gavriliuc, O., Wolke, R., Granert, O., Deuschl, G., & Margraf, N. G. (2020). Quantitative assessment of posture in healthy controls and patients with Parkinson's disease. *Parkinsonism Relat Disord, 76*, 85-90. doi:10.1016/j.parkreldis.2020.01.012
